# Supplementary figures and images for: Identification of Putative Quantitative Trait Loci for Improved Seed Oil Quality in Peanuts
Source: Genes (Basel). 2024 Jan 5;15(1):75. doi: 10.3390/genes15010075 (PMC10815147; doi:10.3390/genes15010075)

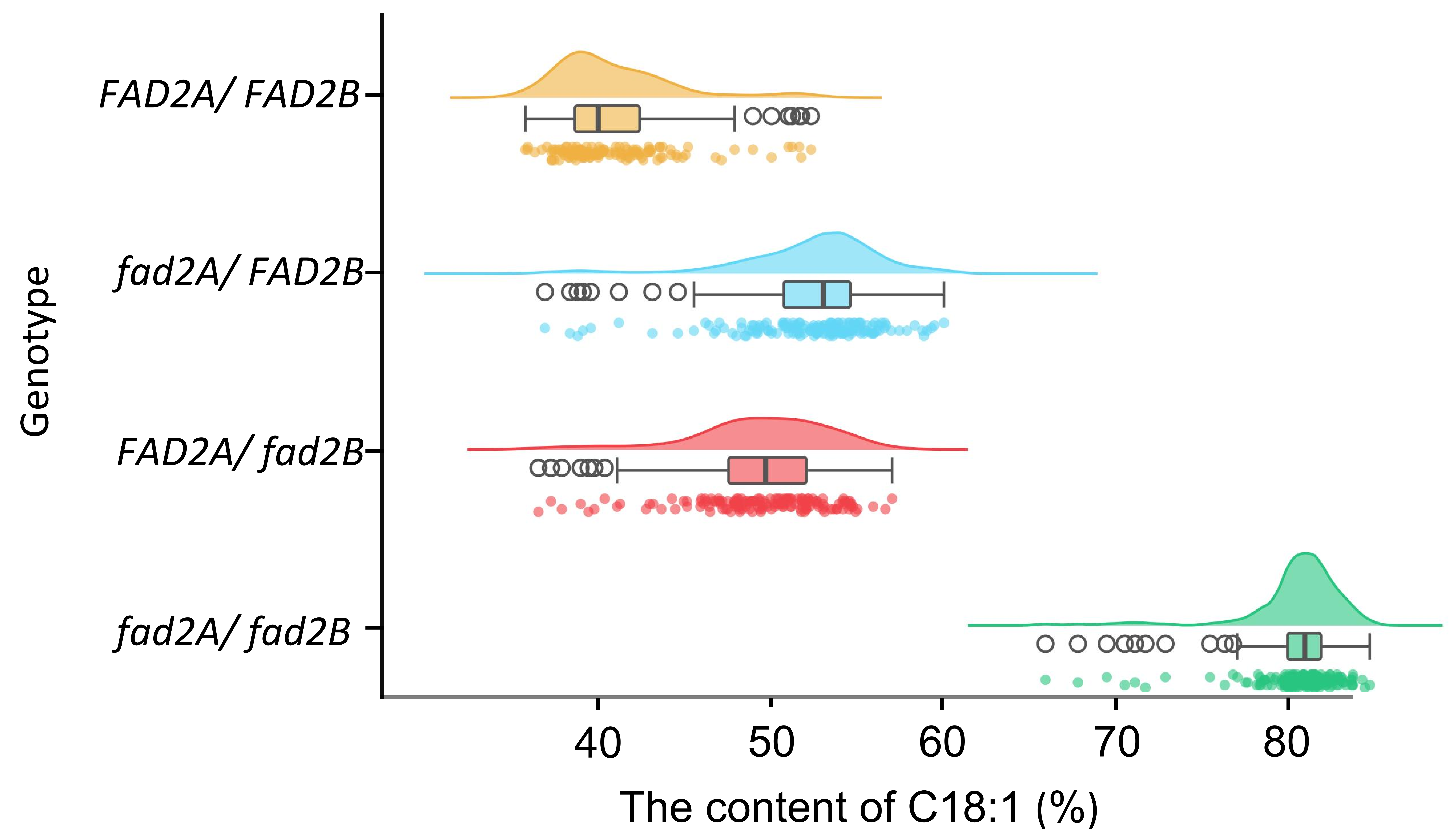

Supplement: Supplementary file 1 [file genes-15-00075-s001.zip › Figure S1.jpg]
